# The Waukesha Medicinal Spring

**Published:** 1869-04-01

**Authors:** 


### CORRESPONDENCE.

### *The Waukesha Medicinal Spring.*

EDITOR CHICAGO MEDICAL JOURNAL:

Allow me to communicate what I know personally regarding the Medicinal Spring recently discovered by accident at Waukesha, as I think it will be of peculiar interest to your readers.

The following is the way in which the spring was discovered: Col. Dunbar, a wealthy citizen of New York, being on a visit to his sister-in-law, in Waukesha, was walking in the outskirts of the village, one day, in company with some friends. He had been laboring under *diabetes saccharum* for some fifteen months previous, and had availed himself of the best medical skill in both Europe and America without obtaining any relief. He drank several gallons of water daily, and passed about an equal quantity, being obliged to frequently rise during the night. His water was repeatedly tested by Dr. Parker, in New York, and found loaded with sugar, and where it dribbled away, it stiffened his clothing so that the cloth would break like pasteboard. His thirst was constant and intense, so much so that he was in the habit of carrying a bottle of water in his pocket, to quench it. He was much reduced in flesh and strength, had a very bad color, and his gums had the appearance of those of a person salivated. On the occasion referred to he became so parched with thirst that he could scarcely speak. His friends said: "By the way, there is a spring near by; we'll get a glass and go down there and let you drink." This they did. The Colonel felt a peculiar relief immediately—unlike that afforded by common water. I should remark that this spring had been known to the villagers for thirty years; the boys often used to catch "minnies" out of it for fish-bait, but no one ever suspected that it contained medicinal virtues.

After lying awhile under the shade of an oak tree which stands near the spring, the Col. went back and drank freely of the water again. He then went home perspiring freely.

Strange to say that from the first taste of the water his symptoms began to abate. He felt so much relieved next morning that he visited the spring early, and drank freely again, and continued to do so, until he recovered entirely. Within twenty-fours, the fierce thirst had left him, and he was no longer obliged to rise during the night. Believing his cure permanent, the Col. returned to his old mode of high living, wine, etc., and his complaint returned. He again had recourse to the water, by the advice of Dr. Parker, and was again cured within twenty-four hours. This winter, being in Washington, and living high, it returned a third time, and he again wrote Dr. Parker. The Doctor's prescription was, "Drink the water again." A few weeks since, the Col. arrived in Waukesha to follow this advice, and, as before, received immediate relief. He has gained five pounds in flesh, and looks as well as any man I ever saw, and has not a particle of his old trouble. I know Col. Dunbar personally, and write of what I know. He is a man weighing 235 pounds, stout and fleshy. On a healthy person, the water is, I judge from my own experience, decidedly diuretic; but those who have used it for diabetes, with great unanimity, declare that "the more they drink the less they urinate," it seeming to have an astringent effect on the kidneys.

The spring itself bubbles up under a hillside, among sand and limestone rock. The water is as clear as air, and tastes like pure spring water—has no other perceptible taste—but is so impregnated with salts of some kind that the pond into which the rivulet from the spring empties itself, which is about seventy-five feet in circumference, *never freezes* during the coldest weather, although fully exposed and unsheltered. The spring water is about 50° or 60° Fahr.—being that of ordinary well water. I have visited the spring, and noticed several small fish in it. The water is pleasant to use, and is used for table and drinking purposes entirely by Miss Clark (the Colonel's sister-in-law) and her family. It is the middle and largest one of three springs, within thirty feet of each other, all, apparently, having the same origin, and are probably of similar properties, although the water of the other two have never been tried. If there is any iron in the water, it must be the merest trace, as it presents none of the characteristics of the chalybeates.

If drank freely before breakfast, the water is mildly aperient, and is said to be one of the finest regulators of the bowels ever tried, and a sovereign remedy for habitual costiveness. Some who have used it complain of its causing vomiting. I have drank as much as a quart of it within an hour, without noticing any special effect, except a sharpening of the appetite. An analysis, I understand, has been made of it, for Dr. Parker, but I have not seen it. When boiled down, it leaves a thick, syrupy substance at the bottom of the cauldron.

It has performed three other cures, all of whom I have seen and conversed with, scarcely less remarkable. The Colonel's sister-in-law had labored for years under a distressing "*gravel.*" The water has affected a complete cure of this difficulty in her case. Mr. J. J. Clark had the appearance of a person with confirmed tuberculosis. It was generally conceded that he would not live three months, as his sister had died of the same disease. He was induced to drink the water, although he had no faith in its efficacy. The result was most marvelous. The cough left him, his appetite, digestion, and spirits returned; he fleshed up, all his pants "got too small for him," and, to-day, a more healthy looking man I never saw, and he has no cough, and says he feels as well as he ever did in his life.

The last day I was at the spring, Mr. Kimball, an old and respected citizen, living near the village, and who, like most old men, being troubled with his water-works for years—his trouble being similar to Col. Dunbar's, so far as symptoms went, although whether the urine was saccharine or not I do not know—drank of the water, obtained speedy relief, and is now entirely free of the difficulty. He was, in fact, jumping around, expressing his satisfaction and sense of relief in the most lively manner.

Here, then, are at least four remarkable and well-authenticated cures, all the parties being residents of the place, and their cases well known to all the citizens, and they are all .ready and eager to tell how they were cured. There must certainly be some wonderful virtue in the water; and if it should prove as beneficial in all cases of *diabetes* alone, as it has in Col. Dunbar's and Mr. Kimball's, it will be a boon to suffering humanity, the value of which cannot be overestimated, for this disease has, heretofore, baffled all known remedies. But what shall we say of it if it should prove equally beneficial in all derangements of the kidneys, rheumatic and gouty complaints? And what a tide of pil grims from every part of the world will seek the healing pool if all cases of tuberculosis similar to Mr. Clark's should be cured by its use?

At present the spring is free to all, and, perhaps, always will remain so; the Colonel and his sister-in-law, who have purchased the ground, being persons of enlarged and liberal views on such matters. The supply of water is inexhaustible, and all who will, may drink freely, without money and without price. All that is needed is a good hotel to accommodate those visiting the waters—and this the Colonel assures me he intends to erect the coming summer.

The water deserves a thorough analysis, and is now being tried by Dr. Parker, of New York, in the hospital under his charge; and also by a number of parties—invalids—on the ground. F. W. Williams, M.D.

---

*March 16th, 1869.*

J. Adams Allen, M.D.:

Dear Sir: Enclosed find balance for the Journal as per bill. If it is not presuming too much upon your good nature, I would like to mention a remarkable cure of epilepsy which occurred in the limits of my practice. The patient, a girl of nineteen years, had been afflicted from childhood with epilepsy, the paroxysms recurring every two or three weeks, and continuing from six to fifteen hours each time, until the intellect was almost destroyed. About ten months ago she became pregnant, and the paroxysms became less frequent, and at the end of two months entirely cured. I attended her during confinement, and, contrary to my expectations, there was no appearance of convulsions during, or following, labor; nor has there been any symptoms of them since. Is the man who effected the cure a "Quack" or a "Regular?"

